# Atypical Spindle Cell Lipomatous Tumor: A Scoping Review of Current Insights Into Diagnosis, Pathogenesis, and Management

**DOI:** 10.7759/cureus.98997

**Published:** 2025-12-11

**Authors:** Noor Chughtai, Jordan Shelestak, Cortland Brown, Jared Nichols

**Affiliations:** 1 College of Osteopathic Medicine, Kansas City University, Joplin, USA; 2 Osteopathic Manipulative Medicine, Kansas City University, Joplin, USA

**Keywords:** diagnostic pathology, immunohistochemistry, lipomatous neoplasm, soft tissue tumor, spindle cell tumor

## Abstract

Atypical spindle cell/pleomorphic lipomatous tumor (ASPLT) is a recently recognized benign adipocytic neoplasm, distinguished by specific histopathological and immunohistochemical features. Although traditionally classified as a tumor of the limbs and limb girdles, emerging evidence suggests a broader anatomic distribution. A systematic review of the literature was conducted using PubMed to identify ASPLT case reports published within the past decade. Cases were included if they had a confirmed ASPLT diagnosis, while reports describing unrelated neoplasms or limited by language barriers were excluded. Thirteen cases met the inclusion criteria. Data on patient demographics, tumor characteristics, immunohistochemical profiles, and diagnostic methodologies were extracted and analyzed.

CD34 expression was observed in 90% of cases, while desmin staining was inconsistently reported and lacked clear diagnostic value. Notably, 69% of cases omitted testing for Rb protein loss, a key marker in ASPLT classification. Unexpected MDM2 positivity was identified in 44% of tested cases, with one demonstrating gene amplification, suggesting potential overlap with atypical lipomatous tumor/well-differentiated liposarcoma (ALT/WDL). Additionally, ASPLT occurred more frequently in axial locations than previously recognized, with eight cases located in the head, neck, and thoraco-lumbar regions. These findings emphasize the need for standardized diagnostic protocols, including routine Rb evaluation and MDM2 analysis, to ensure accurate classification. Future studies should explore the significance of MDM2 expression in ASPLT and reassess its anatomic predilection to refine diagnostic criteria and clinical management strategies.

## Introduction and background

Atypical spindle cell/pleomorphic lipomatous tumor (ASPLT) is a rare, benign adipocytic neoplasm that was officially recognized by the World Health Organization (WHO) in its 2020 classification of soft tissue tumors [1]. It has been recently characterized and added to the spectrum of lipomatous tumors. ASPLT predominantly affects adults, with a slight male predominance, and typically presents as a slow-growing, painless mass in the subcutaneous tissues of the limbs and limb girdles [2]. While initially mistaken for other lipomatous or spindle cell tumors, ASPLT has been recognized as a distinct entity based on its unique histopathological and immunohistochemical characteristics [1].

Because ASPLT is a recently classified entity with evolving diagnostic criteria, the available literature is limited and often heterogeneous. This review focuses on uncommon anatomic sites and presentations that fall outside the classic WHO-defined pattern, which naturally introduces variability in reported histologic features, immunoprofiles, and clinical behavior. Accordingly, the objective is not to overstate diagnostic distinctions or imply uniformity across cases, but to synthesize existing evidence, acknowledge areas of inconsistency, and outline aspects of atypical spindle cell lipomatous tumor (ASCLT) that remain uncertain or subject to ongoing debate.

Histologically, ASPLTs exhibit a diverse cellular composition, including atypical spindle cells, mature adipocytes, lipoblasts, floret-like multinucleated giant cells, and pleomorphic cells, set within a stroma that ranges from myxoid to collagenous. Immunohistochemistry plays a pivotal role in diagnosis, with tumor cells typically expressing CD34, which highlights fibroblastic and spindle cell components, and desmin, indicative of myogenic differentiation, while showing variable S100 expression limited to adipocytic or neural elements [3]. A distinguishing feature is the loss of retinoblastoma protein (pRb) due to RB1 deletion, which aids in differentiation from similar-appearing neoplasms [2]. In contrast, MDM2 and CDK4, markers associated with well-differentiated liposarcoma, are not amplified in ASPLT, further supporting diagnostic distinction [4].

Differentiating ASPLT from other soft tissue tumors is crucial for appropriate management. One of the primary differentials is spindle cell lipoma (SCL), which, like ASPLT, expresses CD34 but lacks desmin and retains pRb expression [4]. Pleomorphic lipoma (PL) similarly expresses CD34 and retains pRb, making immunohistochemistry a key distinguishing factor. Well-differentiated liposarcoma (WDL) or atypical lipomatous tumor (ALT) can mimic ASPLT but are characterized by MDM2 and CDK4 positivity, markers that are absent in ASPLT [5]. Mammary type myofibroblastoma (MFB), another histologically similar entity, expresses both desmin and CD34 while also exhibiting pRb loss, necessitating careful clinical and histological correlation for accurate diagnosis [6]. Lipofibromatosis-like neural tumor (LPF-NT) presents with spindle cells infiltrating adipose tissue and expresses both S100 and CD34 but is negative for SOX10, further distinguishing it from ASPLT [7].

Clinically, ASPLTs are considered benign neoplasms with a low but notable risk of local recurrence. There is no documented risk of metastasis, and complete surgical excision with clear margins is the treatment of choice [8]. Due to the potential for local recurrence, regular follow-up is recommended to monitor for any signs of regrowth. In conclusion, ASPLT is a distinct benign adipocytic tumor with unique histological and immunohistochemical features. Accurate diagnosis necessitates a comprehensive evaluation to differentiate it from other similar-appearing neoplasms, ensuring appropriate management and prognostication.

Objective

The objective of this systematic review is to examine ASPLTs and highlight the importance of recognizing and diagnosing these tumors, even when they present in uncommon anatomical locations. While ASPLTs are most frequently identified in the limbs and limb girdles, there have been cases of occurrence in less typical sites, which may lead to misdiagnosis or underdiagnosis. By bringing awareness to these variations, this paper emphasizes the necessity of thorough histopathological evaluation and immunohistochemical staining in all suspected cases. Given the distinct immunoprofile of ASPLTs, including CD34 and desmin positivity with loss of pRb expression, routine staining and molecular analysis should be performed to confirm the diagnosis and differentiate ASPLTs from histologically similar tumors. Increasing awareness of these diagnostic challenges can improve early detection and ensure accurate classification, ultimately guiding appropriate clinical management.

Methods

A systematic search was conducted by three researchers using PubMed to identify case reports on ASCLT. The search utilized the key term "atypical spindle cell lipomatous tumor" to ensure comprehensive retrieval of relevant literature. Inclusion criteria was case reports published in English from 2015-2025. Exclusion criteria included papers written in a language other than English and studies where the final diagnosis was spindle cell lipoma, spindle cell liposarcoma, or another unrelated tumor. Although these entities share histopathological similarities with ASCLT, they are distinct diagnoses and were therefore excluded from the analysis. The search initially identified 30 cases. After applying the exclusion criteria, seven studies were excluded for discussing spindle cell lipomas, nine for focusing on liposarcomas, and one for describing a typical carcinoid tumor. This left a final dataset of 13 studies for qualitative analysis.

**Figure 1:**
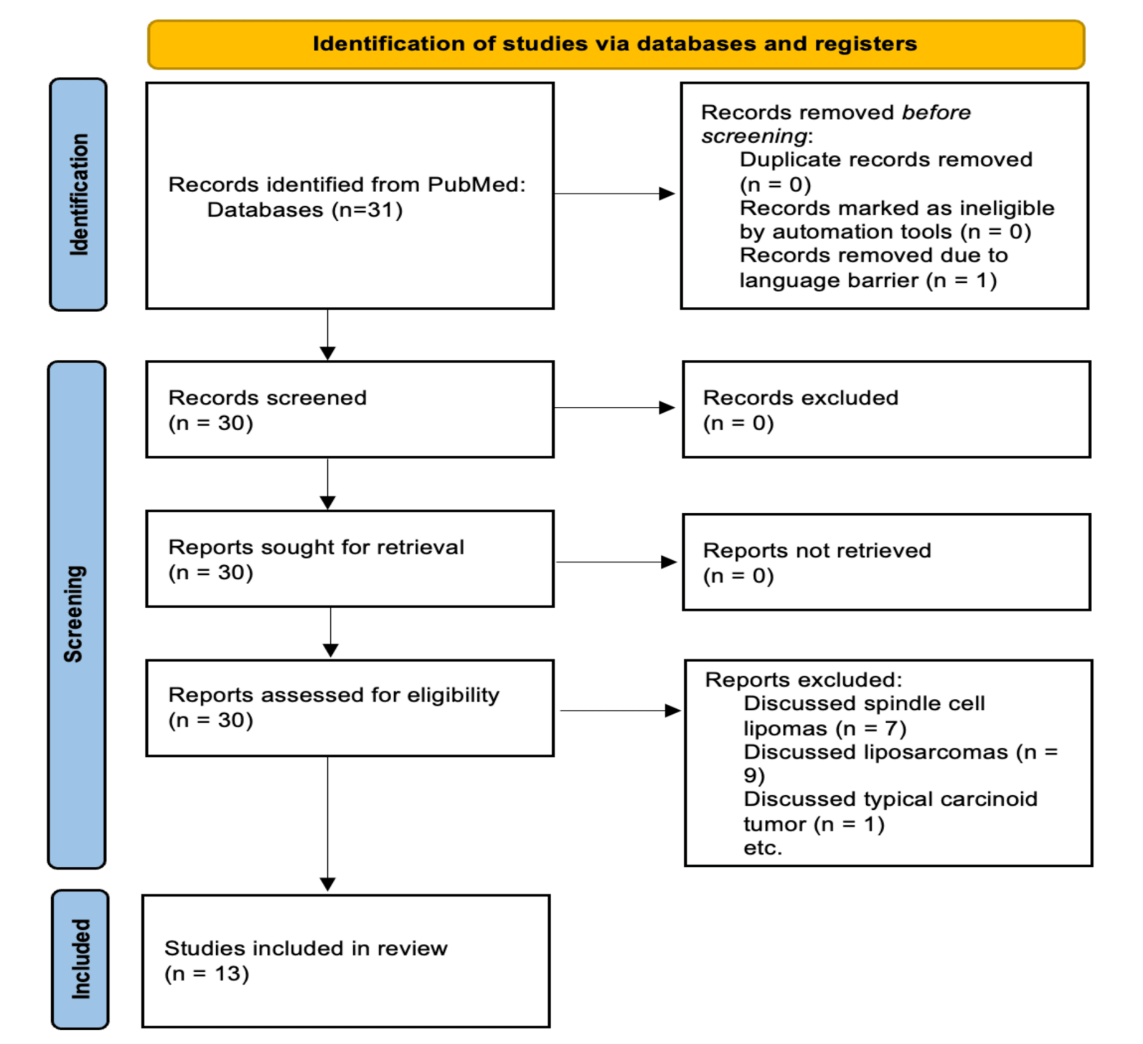
Preferred Reporting Items for Systematic Reviews and Meta-Analyses (PRISMA) Diagram

Data were extracted from each study, including patient demographics (age, sex, clinical presentation), tumor characteristics (location, size, histological features), immunohistochemical (IHC) markers used, and final pathology conclusions. The Joanna Briggs Institute (JBI) Critical Appraisal Checklist for Case Reports was used to assess the methodological quality of the included studies. Studies were evaluated based on the clarity of patient demographics, description of the diagnostic approach including IHC markers, and completeness of follow-up and final diagnosis confirmation. Articles meeting at least six out of eight criteria on the checklist were considered of high methodological quality. The study selection process is summarized in a Preferred Reporting Items for Systematic Reviews and Meta-Analyses (PRISMA) flow diagram (Figure 1).

## Review

Results

A case-by-case summary of the findings can be found in Table 1, with each case report being assigned the corresponding number as it appears in the references section for ease. The age and gender, anatomic location, and tumor marker findings are listed for each case report. The following tables are comprised of the aggregated values in order to extract potential clinical/pathological relevance as it relates to atypical spindle cell lipomatous tumors.

**Table 1:** Summary of analyzed case reports of ASCLT with age, gender, location, and markers ASCLT, atypical spindle cell lipomatous tumor; MDM2, murine double minute 2 homolog; Rb, retinoblastoma gene.

Case Number	Author	Age and Gender	Location of ASCLT	CD34	MDM2	MDM2 amplification	Desmin	CDK4	Loss of RB1	S100
[1]	Graja et al., 2022	77 y.o. female	Tongue	✓	✓	X	X	X	-	-
[5]	Yoshia et al., 2020	74 y.o. male	Tongue	✓	✓	-	-	X	-	-
[7]	Rodrigues et al., 2021	58 y.o. male	Right groin	✓	✓	-	-	X	-	✓
[9]	Ahn SH et al., 2023	57 y.o. male	Orbit	✓	X	-	-	X	-	-
[10]	Al-Kadi et al., 2022	81 y.o. male	Hypopharynx	✓	✓	✓	-	-	-	-
[11]	Bae et al., 2022	18 y.o. female	Retroperitoneum	✓	X	X	✓	-	X	✓
[12]	Cheng et al., 2023	38 y.o. male	Lumbar paraspinal region	✓	X	-	X	X	✓	X
[13]	Ichikawa et al., 2022	64 y.o. male	Right buttock	✓	X	X	X	-	✓	✓
[14]	Lugwaja et al., 2023	52 y.o. male	Retroperitoneum	✓	-	-	-	-	-	-
[15]	Lyatoshinsky et al., 2023	79 y.o. male	Seminal vesicle	✓	-	-	-	-	-	-
[16]	Tchack et al., 2021	74 y.o. male	Right superior posterior thorax	X	X	-	X	X	✓	✓
[17]	Bhattarai et al., 2022	60 y.o. male	Anterior abdominal wall	-	-	-	-	-	-	-
[18]	Iseed et al., 2024	13 y.o. male	Vastus lateralis muscle	-	-	-	-	-	-	-

The aggregated IHC results are summarized in Table 2. Each marker is listed with the total number of cases that tested positive, negative, or were not assessed. CD34 was the most frequently evaluated marker, performed in all but two case reports. Among those tested, 10 of 11 cases (90.9%) were CD34 positive. Other markers were assessed less consistently, and only S100 demonstrated a positivity rate above 50%, with four of five cases (80%) testing positive.

**Table 2:** Summary of IHC markers in reported ASCLT cases IHC, immunohistochemistry; ASCLT, atypical spindle cell lipomatous tumor

Immunohistochemistry	Cases Positive	Cases Negative	Not Tested
CD34	10 (76%)	1 (7%)	2 (15%)
MDM2	4 (30%)	5 (38%)	4 (30%)
MDM2 Amplification	1 (7%)	3 (23%)	9 (69%)
Desmin	1 (7%)	4 (30%)	8 (61%)
CDK4	0 (0%)	6 (46%)	7 (54%)
RB1	1 (7%)	3 (23%)	9 (69%)
S100	4 (30%)	1 (7%)	8 (62%)

The demographics of ASCLT are summarized in Table 3. While the median age at diagnosis was 60 years, cases ranged widely from 13 to 81 years. The occurrence in a 13-year-old highlights the importance of carefully evaluating the diagnostic criteria for ASCLT, as the tumor is traditionally associated with middle-aged to older adults. Regarding sex distribution, the tumor showed a striking male predominance, with a reported male-to-female ratio of 12:1.

**Table 3:** Summary of common demographic patterns in reported ASCLT cases ASCLT, atypical spindle cell lipomatous tumor

Demographics	Years
Median Age	60
Range	(13-81)
Male:Female	12:1

The anatomic distribution of reported ASCLT cases is outlined in Table 4. Although ASCLT is classically described as arising in the extremities and limb girdles, a substantial number of the 13 cases were located in the axial skeleton and trunk. These findings suggest that the traditional understanding of ASCLT localization may be more variable than previously recognized.

**Table 4:** Summary of anatomic patterns in reported ASCLT cases ASCLT, atypical spindle cell lipomatous tumor

Anatomic Location	Cases
Superficial head/neck	1
Tongue	2
Hypopharynx	1
Upper back	1
Lower back	1
Lower limb	2
Groin	2
Abdominal wall	1
Retroperitoneal	2

Of the 13 cases examined, nine discussed follow-up periods of at least six to eight weeks post-surgery. These findings are found in Table 5. Of the nine, eight showed no evidence of reoccurrence with follow-up. This is consistent with ASCLT's relatively benign presentation as a painless, well-circumscribed mass. Only one case discussed a single, local reoccurrence, while no cases discussed multiple reoccurrences or metastasis. 

**Table 5:** Summary of clinical follow-up and outcomes in reported ASCLT cases ASCLT, atypical spindle cell lipomatous tumor

Follow-Up Post-Excision	Number of Cases (Percent of Cases Evaluated Post-op)
No evidence of reoccurrence	8 (88%)
Single, local reoccurrence	1 (11%)
Multiple, local reoccurrences	0 (0%)
Metastasis	0 (0%)

The exact sizing of each tumor as measured and presented in each case report is listed in centimeters in Table 6. Volume was calculated by treating the tumors as an ellipsoid, where V=6π​×L×W×H in cm³. The smallest of tumors was found to be 0.91 cm³, whereas the largest was reported to be 5497.79 cm³. This shows the large range of sizing possible in ASCLT and the potential for obstruction and mass effect of these tumors.

**Table 6:** Reported tumor dimensions in ASCLT cases V, volume; L, length; W, width; H, height; ASCLT, atypical spindle cell lipomatous tumor

Case	Length (cm)	Width (cm)	Height (cm)	Volume (cm³)
[1]	1.8	1.2	0.8	0.9047786842
[5]	-	-	-	-
[7]	-	-	-	-
[9]	-	-	-	-
[10]	2.5	2	2	5.235987756
[11]	38	24	11.5	5491.503958
[12]	11.5	2	2	24.08554368
[13]	16	15	12	1507.964474
[14]	35	25	12	5497.787144
[15]	7	6	6	131.9468915
[16]	-	-	-	-
[17]	10	10	1	52.35987756
[18]	10.5	7.1	11.4	444.9908914

**Figure 2:**
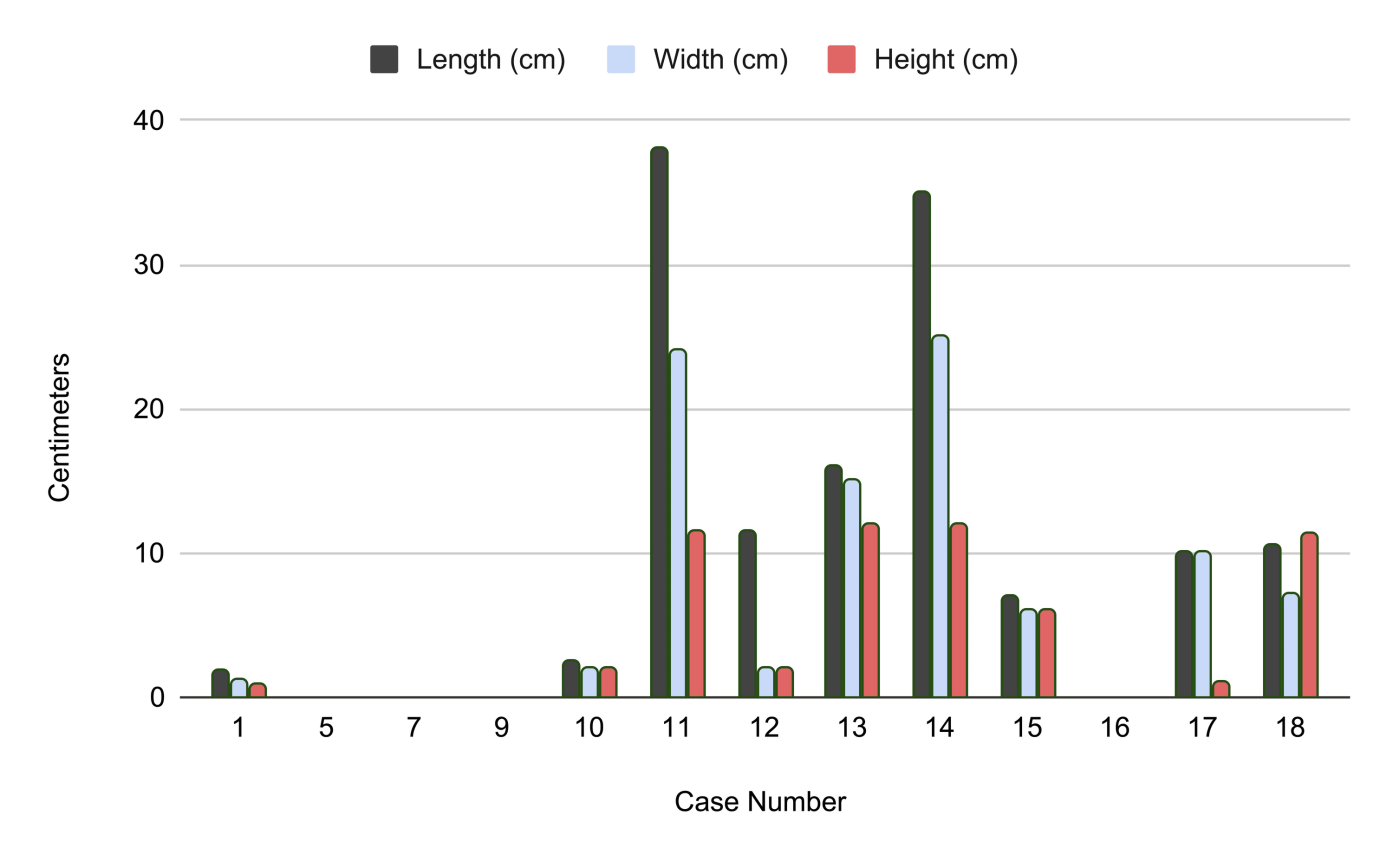
Comparison of tumor sizes in reported ASCLT cases ASCLT, atypical spindle cell lipomatous tumor

Figure 2 provides a graphical representation of tumor dimensions (length, width, and height in centimeters), complementing the data presented in Table 6.

Discussion

Interpretation of IHC Findings and Their Diagnostic Utility

The majority of cases in this review that included CD34 testing demonstrated positivity (91%), aligning with the established immunophenotype of ASPLT. While this supports the usefulness of CD34 in distinguishing ASPLT from histologic mimics such as WDL and ALT, the small sample size and inconsistent reporting across cases limit the strength of this observation. These findings should therefore be interpreted as descriptive rather than definitive patterns.

Desmin positivity has traditionally been described as a characteristic feature of ASPLT in prior literature; however, our dataset raises questions about the consistency of this marker in diagnosis. Of the 13 cases reviewed, only five (38%) cases tested for desmin, and among those, only one case demonstrated positivity. Given the small number of cases tested, it is unclear whether desmin negativity represents true variability within ASPLT or if desmin positivity should no longer be considered a defining feature. The limited and inconsistent use of desmin across cases prevents conclusions about its diagnostic utility. 

Similarly, although loss of Rb protein is considered a critical diagnostic marker for ASPLT, Rb status was only reported in four cases. Three of these showed Rb loss, aligning with prior descriptions, while one case retained Rb expression. Notably, this case occurred in a younger patient, which is uncommon in ASPLT. This raises curiosity about potential age-related variations in Rb status or alternative molecular pathways in younger patients, but given the very small number of tested cases, it is not possible to determine whether this represents a molecular subset or diagnostic uncertainty. The lack of testing highlights a broader limitation in the available literature and emphasizes the need for standardized inclusion of Rb in future diagnostic panels. 

MDM2 testing was also performed inconsistently. Of the nine cases reporting this marker, four demonstrated unexpected positivity, including one with confirmed MDM2 gene amplification. While MDM2 amplification is generally associated with ALT/WDL rather than ASPLT, the limited number of cases and the variability in testing methods across reports prevent any firm conclusions. These findings may reflect diagnostic pitfalls or rare exceptions, but they may also stem from misclassification or incomplete evaluation. Larger and systematically studied cohorts will be needed to clarify the significance of MDM2 expression in tumors resembling ASPLT. 

A notable limitation in our dataset is that two cases did not report any IHC findings, leaving their classification as ASPLT largely unsubstantiated. Given that ASPLT is defined by a certain immunophenotype - CD34 and desmin positivity with Rb protein loss - the absence of IHC data raises concerns about the reliability of these diagnoses and limits their value in refining our understanding of the tumor’s defining features. Also, it highlights the broader issue of inconsistent reporting in literature and complicates efforts to establish standardized diagnostic criteria. Without a complete IHC profile, distinguishing ASPLT from histologic mimics becomes challenging. These gaps emphasize the need for reporting standards.

Reevaluating the Common Anatomic Location of ASPLT

ASPLT has traditionally been classified as a tumor that predominantly arises in the limbs and limb girdles [19]. In our dataset, however, more cases were located in axial sites, including head and thoracolumbar regions. This observation of more axial cases raises the possibility that ASPLT may occur in a broader range of locations than previously emphasized. The small sample size and reliance on published case reports, which may be subject to reporting bias, prevent any firm conclusions. If future studies corroborate these findings, this may prompt reconsideration of its anatomic distribution. This awareness of axial involvement could potentially aid pathologists and clinicians in accurately diagnosing ASPLT in non-extremity locations. 

Tumor Size Variability 

The cases included in this review showed notable variability in tumor size, with some significantly larger than what has previously been reported for ASPLT. The two largest tumors, both located in the retroperitoneum, averaged an estimated 5,495 cm³ in volume and were the only tumors reported in this location. This raises the possibility that deep-seated tumors may grow larger before detection, though the limited number of cases prevents further interpretation. Conversely, the smallest tumors averaged approximately 3 cm³ and were located in the head and neck region, suggesting a potential, but unproven, anatomic correlation. Two additional head and neck cases did not report tumor size, and four of the 13 total cases lacked size documentation altogether, limiting any meaningful assessment of size-related variability.

Limitations in Current Literature

Despite the insights provided by this review, several limitations must be acknowledged. The small sample size remains a major challenge, as ASPLT is a rare entity with limited published cases. With such a small dataset, it becomes ­­difficult to establish definitive diagnostic and prognostic patterns. Furthermore, inconsistencies in IHC panels across studies complicate direct comparisons and hinder the development of standardized diagnostic criteria. Many case reports lacked essential data, including key IHC markers, tumor size, and patient follow-ups, making it challenging to accurately classify ASPLT and compare findings across studies. Without sufficient follow-ups, it remains unclear whether ASPLT exhibits a risk for local recurrence or malignant transformation, both of which are essential factors in guiding clinical management and patient counseling.

Future Research Directions

Given the limitations in the current literature, several areas of future research are necessary to refine the understanding of ASPLT. First, the development of a standardized IHC panel is crucial to ensure diagnostic accuracy and prevent misclassification. A comprehensive panel including CD34, desmin, Rb protein, and MDM2 should be routinely implemented to distinguish ASPLT from similar soft tissue tumors and to be able to recognize whether a case represents a true subset of ASPLT, shares diagnostic overlap with other tumors, or represents a potential misclassification. Another important avenue of research involves assessing whether Rb1 loss in ASPLT varies by patient age, particularly in younger individuals, as our dataset identified a younger patient with retained Rb expression. This raises questions about age-related variations or alternative molecular mechanisms in younger individuals that warrant further exploration. Finally, epidemiological studies should aim to reassess the anatomic distribution of ASPLT, as our findings question the traditional view that this tumor predominantly arises in the extremities. If additional cases corroborate a predilection for axial locations, it may warrant reconsideration of ASPLT’s anatomic classification. Addressing these research gaps will help establish more reliable diagnostic criteria and improve the clinical understanding of ASPLT.

Clinical Implications for Pathologists and Oncologists 

Our findings emphasize the need for a meticulous diagnostic approach when evaluating lipomatous tumors with atypical features. Pathologists should employ comprehensive IHC panels, including CD34, desmin, and Rb, to differentiate ASPLT from histologic mimics, particularly in cases occurring in rare anatomical locations. Oncologists should be aware of the tumor’s potential to appear in the axial skeleton and should monitor for recurrence despite its benign nature, emphasizing the need for complete excision and long-term surveillance.

The need for consistent diagnostic practices in ASPLT parallels broader trends in clinical medicine. Recent work on artificial intelligence-based diagnostic tools has emphasized the importance of unified protocols and standardized criteria to ensure accuracy and prevent misclassification [20]. Similarly, the reliable identification of ASPLT requires the implementation of standardized IHC panels and structured diagnostic criteria, which would reduce diagnostic variability and enhance clinical confidence. By adopting more uniform practices, pathology and oncology teams can improve diagnostic accuracy and better distinguish ASPLT from morphological molecular mimics. 

## Conclusions

This review highlights key challenges in the diagnosis and characterization of ASPLT, particularly the inconsistencies in IHC testing and the tumor’s broader-than-expected anatomic distribution. While CD34 positivity remains a useful distinguishing marker, the variability in desmin expression and the incomplete assessment of Rb protein loss raise concerns about the reliability of current diagnostic criteria. Additionally, the unexpected presence of MDM2 positivity in some cases suggests potential diagnostic overlap with WDL and ALT, underscoring the need for further molecular studies. Although more axial cases were noted in this small dataset, this observation should be interpreted more cautiously and not assumed to reflect true population-level distribution. Rather than indicating a need for reclassification, these findings simply suggest that ASPLT may present in a wider range of anatomic locations than previously emphasized. Moreover, the lack of standardized reporting on tumor size and follow-up data limits the ability to assess ASPLT’s biological behavior and recurrence potential. To improve diagnostic precision, future studies should focus on the establishment of a standardized IHC panel, the evaluation of age-related differences in Rb expression, and a reassessment of ASPLT’s anatomic distribution using larger datasets. Strengthening these areas of research will help clarify whether the patterns observed in this review represent true trends or are artifacts of limited and inconsistent case reporting, ultimately supporting more clinical and pathological diagnosis.

## References

[1] Graja S, Chaari C, Kammoun C, Zghal M, Dhouib M, Charfi S, Sellami-Boudawara T (2022). Atypical spindle cell lipomatous tumor of the tongue: a rare entity arising in an unusual location. Clin Case Rep.

[2] Anderson WJ, Fletcher CD, Jo VY (2021). Atypical pleomorphic lipomatous tumor: expanding our current understanding in a clinicopathologic analysis of 64 cases. Am J Surg Pathol.

[3] Mukkamalla SK, Lotfollahzadeh S (2025). Breast myofibroblastoma. StatPearls [Internet].

[4] Thway K (2019). Well-differentiated liposarcoma and dedifferentiated liposarcoma: an updated review. Semin Diagn Pathol.

[5] Yoshida Y, Nakabayashi M, Harada Y, Shingu T, Takubo K (2020). A case report of atypical spindle cell lipomatous tumor of the tongue. Yonago Acta Med.

[6] Nishio J, Nakayama S, Chijiiwa Y (2024). Atypical spindle cell/pleomorphic lipomatous tumor: a review and update. Cancers.

[7] Rodrigues E, Cardoso F, Scigliano H, Nora M (2021). An atypical pleomorphic lipomatous tumor presenting as groin mass. Cureus.

[8] Panse G, Reisenbichler E, Snuderl M, Wang WL, Laskin W, Jour G (2021). LMNA-NTRK1 rearranged mesenchymal tumor (lipofibromatosis-like neural tumor) mimicking pigmented dermatofibrosarcoma protuberans. J Cutan Pathol.

[9] Ahn SH, Kim KM, Cho NC, Ahn M (2023). Atypical spindle cell/pleomorphic lipomatous tumor of the orbit: a case report. Korean J Ophthalmol.

[10] Al-Kadi M, AlOtieschan S, Almahdi MJ, AlHajress R (2022). An atypical lipomatous tumor of the hypopharynx: case report. Cureus.

[11] Bae JM, Jung CY, Yun WS, Choi JH (2022). Large retroperitoneal atypical spindle cell lipomatous tumor, an extremely rare neoplasm: a case report. World J Clin Cases.

[12] Cheng YW, Chen YY, Kuo CH, Liao WC, Kwan AL (2023). Lumbar paraspinal atypical spindle cell/pleomorphic lipomatous tumor: a report of a rare case. Clin Case Rep.

[13] Ichikawa J, Kawasaki T, Imada H, Kanno S, Taniguchi N, Ashizawa T, Haro H (2022). Case report: atypical spindle cell/pleomorphic lipomatous tumor masquerading as a myxoid liposarcoma or intramuscular myxoma. Front Oncol.

[14] Lugwaja PW, Ringo Y, Mchele G, Mtaturu G (2023). An extremely rare neoplasm 'atypical spindle cell pleomorphic lipomatous tumor': a case report. J Surg Case Rep.

[15] Lyatoshinsky P, Pratsinis M, Markert E, Schmid HP, Müllhaupt G (2023). Spindle cell/pleomorphic lipoma of the seminal vesicle: first description of a rare benign mesenchymal tumor. Urol Case Rep.

[16] Tchack MS, Broscius M, Reichel M (2021). Primary cutaneous atypical spindle cell lipomatous tumor. Case Rep Pathol.

[17] Bhattarai HB, Chhantyal S, Dahal K (2022). A case report on atypical spindle cell lipomatous tumor: a rare entity. Ann Med Surg (Lond).

[18] Iseed R, Stanford L (2024). Pediatric case of atypical spindle cell/pleomorphic lipomatous tumor within the vastus lateralis muscle. J Surg Case Rep.

[19] Nishio J, Nakayama S, Chijiiwa Y, Koga M, Aoki M (2024). Atypical spindle cell/pleomorphic lipomatous tumor: a review and update. Cancers (Basel).

[20] Ogut E (2025). Artificial intelligence in clinical medicine: challenges across diagnostic imaging, clinical decision support, surgery, pathology, and drug discovery. Clin Pract.

